# Supplementary material for: Diversification of heart progenitor cells by EGF signaling and differential modulation of ETS protein activity
Source: eLife. 2018 Jun 5;7:e32847. doi: 10.7554/eLife.32847 (PMC6033539; doi:10.7554/eLife.32847)
Supplement: Supplementary file 1. — Alleles with cardioblast patterning defects isolated and/or characterized in this study. The table lists the results from the genetic, phenotypic and molecular analysis of the characterized mutants. Indicated nucleotide positions are relative to transcription start site of transcript RA and amino acid positions of protein isoform PA (FB2017_01, released February 14, 2017; D. melanogaster R6.14); * indicates a nonsense mutation, n.d.: not determined. [file elife-32847-supp1.docx]

**Supplementary file 1-Table S1. Alleles with cardioblast patterning defects isolated and/or characterized in this study.**

| **Allele** | **non-complementing alleles and deficiencies** | **gCBs:oCBs ratio** | **nucleotide change** | **amino acid change** | **functional implication of the mutation; comments** |
| --- | --- | --- | --- | --- | --- |
| *bib^S1538^* | *Df(2L)BSC250,* *bib^1^* | = | C9469T | Q516* | truncated protein |
| *Df(2R)edl-S0520* | *Df(2R)Exel7157, Df(2R)edl-L19*  (lethal and  CB pattern defect),  *edl^k06602^*  (reduced viability and  CB pattern defect) | + | deletion | - | complete deletion;  also contains second site mutation in *stj* (lethal with *Df(2R)Exel7128*, ataxic escapers with *stj^k10814^*);  see Figure 4A and Supplementary file 2-Table S2 |
| *edl^k06602^*  (Baker et al., 2001; Török et al., 1993) | *Df(2R)edl-S520*  (reduced viability and CB pattern defect) | + | *P* insert and deletion | - | *edl*-specific deletion removing large portion of 5' coding sequence;  see Figure 4A and Supplementary file 2-Table S2 |
| *Egfr^S0167^* | *Df(2L)Exel6076*, *Egfr^f2^* | - | G33165A | C258Y | missense mutation in furin-like repeat (extracellular domain II) |
| *Egfr^S2145^* | *Df(2L)Exel6076*, *Egfr^f2^* | - | G34331A | C601Y | missense mutation in furin-like repeat (extracellular domain IV) |
| *Egfr^S2307^* | *Df(2L)Exel6076*, *Egfr^f2^* | - | G33210A | C273Y | missense mutation in furin-like repeat (extracellular domain II) |
| *Egfr^S2561^* | *Df(2L)Exel6076*, *Egfr^f2^* | - | T35877A | C1116* | truncation in intracellular tyrosine kinase domain |
| *mam^S0669^* | *mam^8^*, *mam^S4648^* | - | n.d. | n.d. | n.d. |
| *mam^S4648^* | *mam^8^*, *mam^S0669^* | = | n.d. | n.d. | n.d. |
| *mid^S0021^* | *Df(2L)Exel6012*, *mid^1^*, *mid^S2961^* | - | G4255A | - | splice acceptor site mutation in intron 3; truncation in T-box domain; also contains hypomorphic second site mutation in *bib* |
| *mid^S2961^* | *Df(2L)Exel6012*, *mid^1^*, *mid^S0021^* | - | n.d. | n.d. | n.d. |
| *numb^S1342^* | *Df(2L)ED690*, *numb^2^* | + | n.d. | n.d. | n.d. |
| *numb^S3992^* | *Df(2L)ED690*, *numb^2^* | + | n.d. | n.d. | n.d. |
| *numb^S4439^* | *numb^2^*, *numb^S1342^* | + | n.d. | n.d. | n.d. |
| *S^S4550^* | *Df(2L)ED105*, *S^B0453^* | - | C16093T | Q142* | truncation in intracellular domain, loss of transmembrane and lumenal domain |
| *S^B0453^* (Chen et al., 2008) | *Df(2L)ED105*, *S^S4550^* | - | G25248A | E467K | missense mutation in conserved part of lumenal domain |
| *spi^S3384^* | *Df(2L)ED1272*, *Df(2L)ED1303*, *spi^1^* | - | C8676T | R120* | truncation in EGF-like domain likely to result in nonfunctional peptide |
| *spi^1^* (Nüsslein-Volhard et al., 1984) | *spi^S3384^* | - | G8670A | G118R | missense mutation in conserved part of EGF-like domain |
