# Supplementary material for: Diversification of heart progenitor cells by EGF signaling and differential modulation of ETS protein activity
Source: eLife. 2018 Jun 5;7:e32847. doi: 10.7554/eLife.32847 (PMC6033539; doi:10.7554/eLife.32847)
Supplement: Supplementary file 2. — Characterization of edl deletions via PCR. Presence (+) or absence (-) of DNA fragments after PCR reaction including genomic DNA from homozygous S-18a-13b-16c.1 control (WT), Df(2R)edl-S0520, edlk06602 or Df(2R)edl-L19 animals and primer pairs as indicated. CDS: part of coding sequence, TSS: transcription start site, n.d.: not determined. Amplicons are listed in linear order as located on chromosome 2R. * Six additional intronic GEFmeso amplicons were also negative in S0520. [file elife-32847-supp2.docx]

**Supplementary file 2-Table S2. Characterization of *edl* deletions via PCR.**

| **amplicon**  **name** | **size**  **(bp)** | **primer sequences**  **(5' to 3')** | ***WT*** | ***S0520*** | ***k06602*** | ***L19*** |
| --- | --- | --- | --- | --- | --- | --- |
| GEFmeso-1  (CDS) | 473 | GTAAATGGGCTCCTCGCTGAC | + | + | n.d. | n.d. |
|  |  | TGAAGAAGCAACGAAGTAGCACC |  |  |  |  |
| GEFmeso-2  (intron) | 590 | TGGAGAGCCTTAGTAGAGGATTTGC | + | + | + | n.d. |
|  |  | AAACTTGAAGATACGCTGAACTTGC |  |  |  |  |
| GEFmeso-3*  (intron) | 429 | GGAGAAAGTGAATGTCTGCTGACG | + | - | n.d. | n.d. |
|  |  | ATGTGCGGAGGTGCCAACCAGTTC |  |  |  |  |
| GEFmeso-10  (5' exon) | 576 | CGGCTGCTTGATTTACGATTTC | + | - | n.d. | n.d. |
|  |  | CCAACCCCAGAGACAGAAGTCC |  |  |  |  |
| CG10927  (CDS) | 452 | GGAACAACACCTTGAACAGTTTGC | + | - | n.d. | n.d. |
|  |  | GAGTGGTATCCTTCTTGAAATCTTCG |  |  |  |  |
| mRPS28  (CDS) | 510 | CAGGAGACCCAGCAAATGGC | + | - | n.d. | n.d. |
|  |  | TGAATACGGAAACGGCGGAG |  |  |  |  |
| sec6  (CDS) | 661 | AAGAAGGCATCCGTTGAGGC | + | - | n.d. | + |
|  |  | GCGTTATCACCGACTGTTGTAGC |  |  |  |  |
| CG5482  (CDS) | 488 | GCCCAGCATCTTTTGATACATCTC | + | n.d. | n.d. | + |
|  |  | TCACAACCGCCATTCACCTG |  |  |  |  |
| CG30122  (CDS) | 682 | CTTGGTAACGATGTGCGGAAAC | + | n.d. | n.d. | + |
|  |  | AGCGTGAGTCAAAGGCAGCG |  |  |  |  |
| SP2637  (intron3-5) | 580 | AAGCACAGCCAAGTGATTAGCAG | + | n.d. | n.d. | - |
|  |  | CCCCATAAATAAGCGTCGTTTG |  |  |  |  |
| CG33136  (CDS) | 230 | AACTTCTCATCTTCGTCGTCATCG | + | - | + | - |
|  |  | TTGCCACGGGACTCCATTC |  |  |  |  |
| CG33136-up  (upstream *CG33136)* | 319 | TGCGACATCTGTTTGCTGTTACTG | + | - | + | - |
|  |  | TTTTGTGCCTCCTATGAATGGG |  |  |  |  |
| edl-2a  (exon 2, CDS) | 1275 | GTGAAAGACATAAACAGAACATTAGGTCCC | + | - | - | n.d. |
|  |  | CAATCGTGAAAGAGCGAGGGTC |  |  |  |  |
| edl-2b  (within edl-2a) | 201 | TTTCTGTGGCAGTTCGGCGGTG | + | - | - | n.d. |
|  |  | TCATCTACATCCAACTCCTCCGAC |  |  |  |  |
| edl-1  (exon 1, 5'UTR) | 733 | GCTACATACCAACTATAAAAGCCAAGAGTC | + | - | - | - |
|  |  | AACAAAAACCGCTGCACGAG |  |  |  |  |
| edl-up1  (upstream near TSS, spans *edl^k06602^* insertion site) | 563 | GGTAAATCCAGTTTGCCAGTTGC | + | n.d. | - | n.d. |
|  |  | GAAATGCGGGTCTGCATATACAC |  |  |  |  |
| edl-up-5'P  (test for *P* insertion  in *edl^k06602^*) | ca.  350 | CACCCAAGGCTCTGCTCCCACAAT  (=Plac1, binds 5' *P* end) | - | n.d. | + | n.d. |
|  |  | GAAATGCGGGTCTGCATATACAC |  |  |  |  |
| edl-up2  (upstream) | 385 | ATGGCTTGTTTATCAGCAGTTGTCG | + | - | n.d. | n.d. |
|  |  | TTTTGGCTTGGGTTCGTATGTGGAC |  |  |  |  |
| edl-up3  (upstream) | 417 | TGTTTTTCCCCGTTTTCTTCG | + | - | n.d. | n.d. |
|  |  | TTGCCTTGGATGCTACACTCG |  |  |  |  |
| edl-up4  (upstream) | 194 | CGAGTGTAGCATCCAAGGCAAG | + | + | n.d. | n.d. |
|  |  | CACGGAGCATTTCTTCATCGC |  |  |  |  |
| CG15086-1  (5' part of CDS) | 673 | CGTAGCCTTGTCGGTCAAAGAAG | + | + | n.d. | - |
|  |  | TCCTCCTCCAAAAACGAAGAAAG |  |  |  |  |
| CG42855  (CDS/3'UTR) | 696 | AGTGCTGTGCCAGTTCGTGG | + | n.d. | n.d. | - |
|  |  | GCGATTCTGATTGGACAACACC |  |  |  |  |
| Sik3  (CDS) | 621 | CTTCAGTGTATCGCCGCTCAG | + | + | n.d. | + |
|  |  | ATCTGTCCGTTTCCATTCTCCTC |  |  |  |  |
